# IncHI1 plasmids mediated the *tet*(X4) gene spread in *Enterobacteriaceae* in porcine

**DOI:** 10.3389/fmicb.2023.1128905

**Published:** 2023-03-30

**Authors:** Jiangang Ma, Juan Wang, Hua Yang, Mengru Su, Ruichao Li, Li Bai, Jie Feng, Yuting Huang, Zengqi Yang, Biao Tang

**Affiliations:** ^1^State Key Laboratory for Managing Biotic and Chemical Threats to the Quality and Safety of Agro-Products and Institute of Agro-Product Safety and Nutrition, Zhejiang Academy of Agricultural Sciences, Hangzhou, Zhejiang, China; ^2^College of Veterinary Medicine, Northwest A&F University, Yangling, China; ^3^College of Veterinary Medicine, Yangzhou University, Yangzhou, China; ^4^National Health Commission Key Laboratory of Food Safety Risk Assessment, Food Safety Research Unit (2019RU014) of Chinese Academy of Medical Science, China National Center for Food Safety Risk Assessment, Beijing, China

**Keywords:** tigecycline resistance, *tet*(X4), IncHI1, pMLST, *Enterobacteriaceae*

## Abstract

The tigecycline resistance gene *tet*(X4) was widespread in various bacteria. However, limited information about the plasmid harboring the *tet*(X4) gene spread among the different species is available. Here, we investigated the transmission mechanisms of the *tet*(X4) gene spread among bacteria in a pig farm. The *tet*(X4) positive *Escherichia coli*, *Klebsiella pneumoniae*, *Enterobacter cloacae* and *Enterobacter hormaeche* were identified in the same farm. The whole genome sequencing (WGS) analysis showed that the *K. pneumoniae* belonged to ST727 (*n* = 11) and ST3830 (*n* = 1), *E. cloacae* and *E*. *hormaeche* belonged to ST524 (*n* = 1) and ST1862 (*n* = 1). All *tet*(X4) genes were located on the IncHI1 plasmids that could be conjugatively transferred into the recipient *E. coli* C600 at 30°C. Moreover, a fusion plasmid was identified that the IncHI1 plasmid recombined with the IncN plasmid mediated by IS*CR2* during the conjugation from strains B12L to C600 (pB12L-EC-1). The fusion plasmid also has been discovered in a *K. pneumoniae* (K1L) that could provide more opportunities to spread antimicrobial resistance genes. The *tet*(X4) plasmids in these bacteria are derived from the same plasmid with a similar structure. Moreover, all the IncHI1 plasmids harboring the *tet*(X4) gene in GenBank belonged to the pST17, the newly defined pMLST. The antimicrobial susceptibility testing was performed by broth microdilution method showing the transconjugants acquired the most antimicrobial resistance from the donor strains. Taken together, this report provides evidence that IncHI1/pST17 is an important carrier for the *tet*(X4) spread in *Enterobacteriaceae* species, and these transmission mechanisms may perform in the environment.

## Introduction

Tigecycline, a member of tetracyclines, is one of the last-resort antibiotics to treat infections caused by Carbapenem-Resistant *Enterobacteriaceae* (CRE). The tigecycline still exhibits antibacterial activities in the bacteria containing the earlier tetracyclines resistance genes. The plasmid-mediated *tet*(X3) and *tet*(X4) genes conferring tigecycline resistance were discovered in various Gram-negative bacteria, including carbapenem-resistant and colistin-resistant bacterial strains (He T. et al., 2019; Sun et al., 2019; Tang et al., 2022a; Ma et al., 2022b). The *tet*(X) variant-positive isolates from animals, retail meat, and humans have been identified (Zheng et al., 2020; Tang et al., 2021; Umar et al., 2021). Despite the large numbers of *tet*(X) variant genes were discovered, *tet*(X3) and *tet*(X4) were the most popular tigecycline resistance genes, especially *tet*(X4) (Cheng et al., 2020; Guan et al., 2022). The *tet*(X4) gene was widelydetected in *Escherichia coli*, *Klebsiella pneumoniae*, *Aeromonas caviae*, *Citrobacter freundii*, *Acinetobacter indicus*, *Enterobacter cloacae* and so on (Chen et al., 2019; Fang et al., 2020; Li et al., 2021; Zeng et al., 2021; Wu et al., 2022; Zhai et al., 2022).

IncHI plasmid is an important vector for the *tet*(X4) gene, which belongs to the H incompatibility (IncH) group, including IncHI1 to IncHI5 subgroups (Phan and Wain, 2008; Cui et al., 2022). The IncHI1 plasmid is a conjugative plasmid usually larger than 200 kb. IncHI1 plasmid usually contains three replication genes (*repHI1A*, *repHI1B* and *repFIA-like*) (Liang et al., 2018). IncHI1 plasmids are thermosensitive for conjugative transfer, and the efficiency is optimal between 22 and 30°C (Phan and Wain, 2008). It is one of the most common plasmids carrying antimicrobial resistance genes (ARGs) in *Salmonella* (Kubasova et al., 2016). Moreover, IncHI1 plasmids also have been discovered in other *Enterobacteriaceae*, such as *E. coli*, *K. pneumoniae* and *C. freundii* (Dolejska et al., 2013; Hüttener et al., 2019). Significantly, the *tet*(X4) positive IncHI1 plasmids were discovered in several species of *Enterobacteriaceae* (Feng et al., 2022; Gao et al., 2022; Wu et al., 2022).

There are few reports about the mechanism of the *tet*(X4) gene spread between bacterial species. Here, we screened the tigecycline resistance bacteria from a large-scale pig farm in Guangxi province, China. The tigecycline-resistant *E. coli*, *K. pneumoniae*, *E. cloacae* and *Enterobacter hormaechei* were isolated at the same time. The mechanisms of the *tet*(X4) gene transferred among the spaces were unknown. We analyzed the characterization of these strains and compared the ability of conjugative transfer. To the best of our knowledge, this is the first evidence for the IncHI1 and IncHI1-N plasmid harboring the *tet*(X4) gene transferred in several bacteria spp. in a farm.

## Method

### Sample collection and bacterial isolation

Eighty-nine fecal samples were collected from a pig farm in Guangxi province, China, in 2019. These samples were distributed in several stages of the pig’s life, including the piglets, weanling piglets, fattening pigs, and sows. The samples were sent to the laboratory in a cryogenic incubator and screened by the MacConkey agar containing the tigecycline (4 mg/l). The *tet*(X) gene was detected by PCR as the primer (F: 5’-TGGACCCGTTGGACTGACTA-3′, R: 5’-CACTTCTTCTTACCAGGTTC-3′) and sequenced by Sanger Sequencing for the tigecycline resistant strains. Then the *tet*(X)-positive strains were identified by 16S rDNA PCR and sequencing.

### Whole genome sequencing

Whole genome sequencing (WGS) of all isolates was performed using the Illumina HiSeq platform (Yu et al., 2018). The sequences were assembled with SPAdes and analyzed *via* the CGE server.^1^ To further characterize the *tet*(X4) gene in the isolates, three *K. pneumoniae* strains, one *E. cloacae* strain and one *E. hormaechei* were sequenced by the Nanopore MinION platform and assembled by Unicycler. Then the sequence was annotated with the RAST server.^2^ The novel plasmid multilocus sequence typing MLST (pMLST) of IncHI1 was assigned by PubMLST.

### Conjugation testing

The *tet*(X4) positive *K. pneumoniae*, *E. cloacae* and *E. hormaechei* were used as the donor strains, and the *E. coli* C600 (rifampin-resistant) was used as the recipient. In addition, *E. coli* J53 (sodium azide-resistant) was the recipient when the transconjugants used as the donor (Tang et al., 2019, Tang, B. et al., 2020; Lin et al., 2022). The cultures of donor and recipient strains were mixed in fresh LB stationary at 30°C or 37°C overnight. The mixed cultures were collected by centrifugation, then diluted with PBS. The transconjugants were selected by the LB plate containing 4 μg/ml of tigecycline with rifampin (100 μg/ml) at 37°C overnight. Each experiment was repeated three times.

### S1-nuclease digestion pulsed-field gel electrophoresis (S1-PFGE)

S1-PFGE was used to detect plasmid size in strains performed as previously described (Ma et al., 2022a,b; Tang et al., 2022b). In brief, the plugs were made from the fresh cultures embedded in 2% gold agarose and lysed with cell lysis buffer. Then S1 nuclease was used to cut the plugs, and the *Salmonella* H9812 was restricted with *Xba*I as the marker. The plasmids were separated with the CHEF Mapper XA system.

### Antimicrobial susceptibility testing

The minimum inhibitory concentrations (MICs) for 13 antimicrobial agents (ampicillin (AMP), amoxicillin-clavulanate (A/C), gentamicin (GEN), florfenicol (FFC), tetracycline (TET), tigecycline (TIG), ceftiofur (CEF), ceftazidime (CAZ), enrofloxacin (ENR), sulfisoxazole (SUL), imipenem (IMP), meropenem (MEM), colistin (COL)) were determined by the broth microdilution method according to the Clinical and Laboratory Standards Institute (CLSI) as previously described (Ma et al., 2020; Tang et al., 2022a). The wild strains and transconjugants were tested.

## Result

### Prevalence of *tet*(X4)-positive isolates

A total of twelve (13.48%) *K. pneumoniae*, one (1.12%) *E. cloacae* and one (1.12%) *E. hormaechei* were screened by MacConkey agar with tigecycline (4 mg/l) from 89 swine feces samples in Guangxi Province in China. Furthermore, 26 (29.21%) *tet*(X4)-positive *E. coli* had been isolated and reported in a previous study (Feng et al., 2022). All of these strains carrying the *tet*(X4) genes were identified by PCR and sequencing.

### Genomic epidemiology of *tet*(X4)-positive *Klebsiella pneumoniae*, *Enterobacter cloacae* and *Enterobacter hormaechei*

The Multi-Locus Sequence Typing (MLST) analysis showed that 12 isolates of *K. pneumoniae* belong to ST727 (11/12) and ST3830 (1/12), the *E. cloacae* belong to ST524 and the *E. hormaechei* belongs to novel type (ST1862), respectively. The *K. pneumoniae* showed a clone spread with the ST727 type.

**Figure 1:**
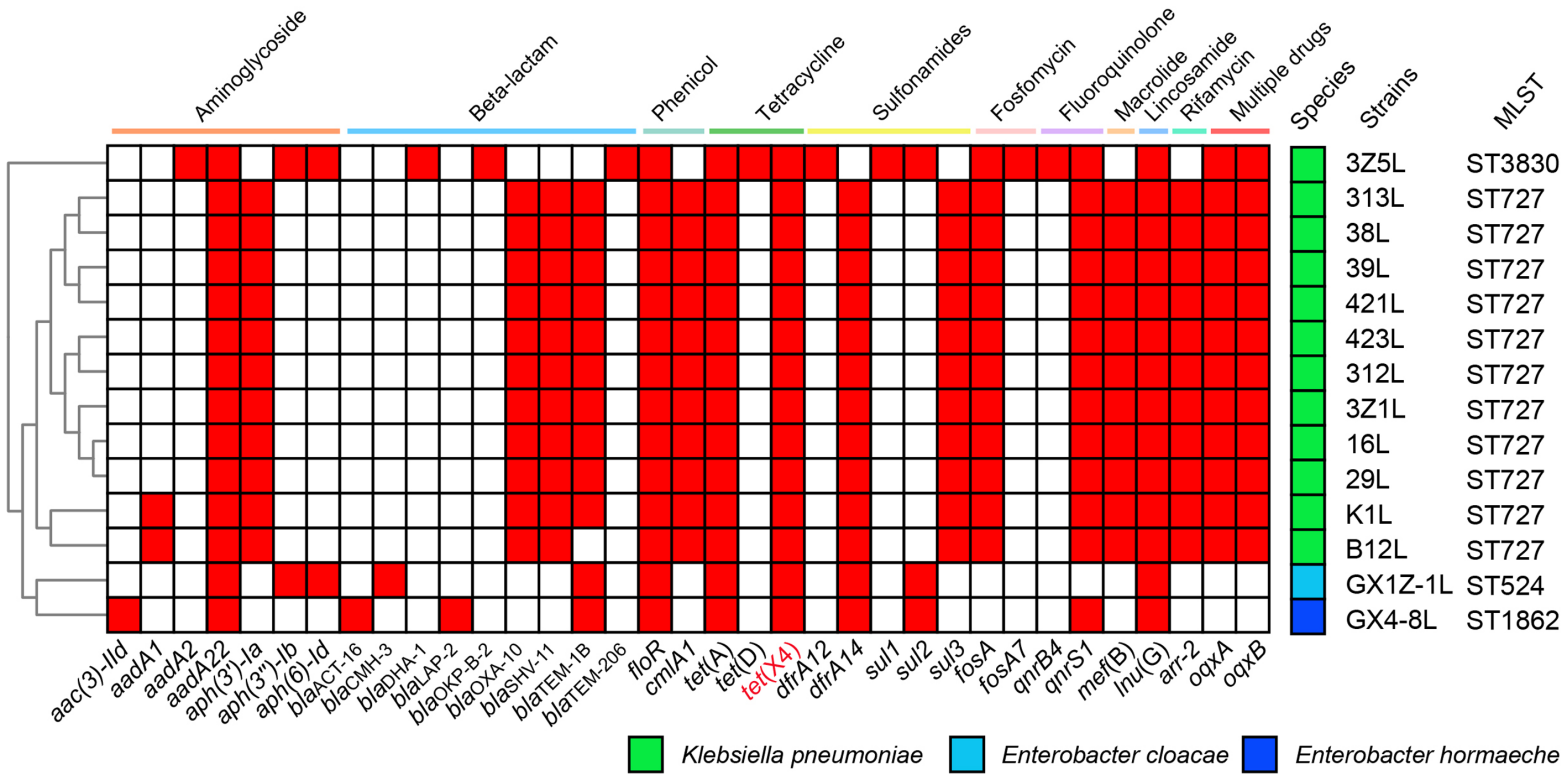
The heat map of antimicrobial resistance genes of 14 strains in this study. The positive genes in strains are marked with the red box. The names of genes are labeled below the heat map.

The ARGs prediction showed the strains have various resistance genes, including *bla*_TEM-1_, *bla*_OXA-10_, *bla*_SHV-11_, *floR*, *cmlA1*, *fosA*, *mef*(B), *oqxA*, *oqxB*, *qnrS1*, *sul3*, *tet*(A), *tet*(X4), *aadA12*, *aph(3*′*)-Ia*, *dfrA14*, *arr-2* and so on (Figure 1). The *K. pneumoniae* strains 16 l, 29 l, 38 l, 39 l, 312 l, 313 l, 421 l, 423 l, K1L, 3Z1L and B12L belonged to ST727 that have a similar ARGs. They were different from the *K. pneumoniae* 3Z5L (ST3830), *E. cloacae* GX1Z-1 l (ST524) and *E. hormaechei* GX4-8 l (ST1862).

### Genetic structures of tet(X4)-positive plasmids

**Figure 2:**
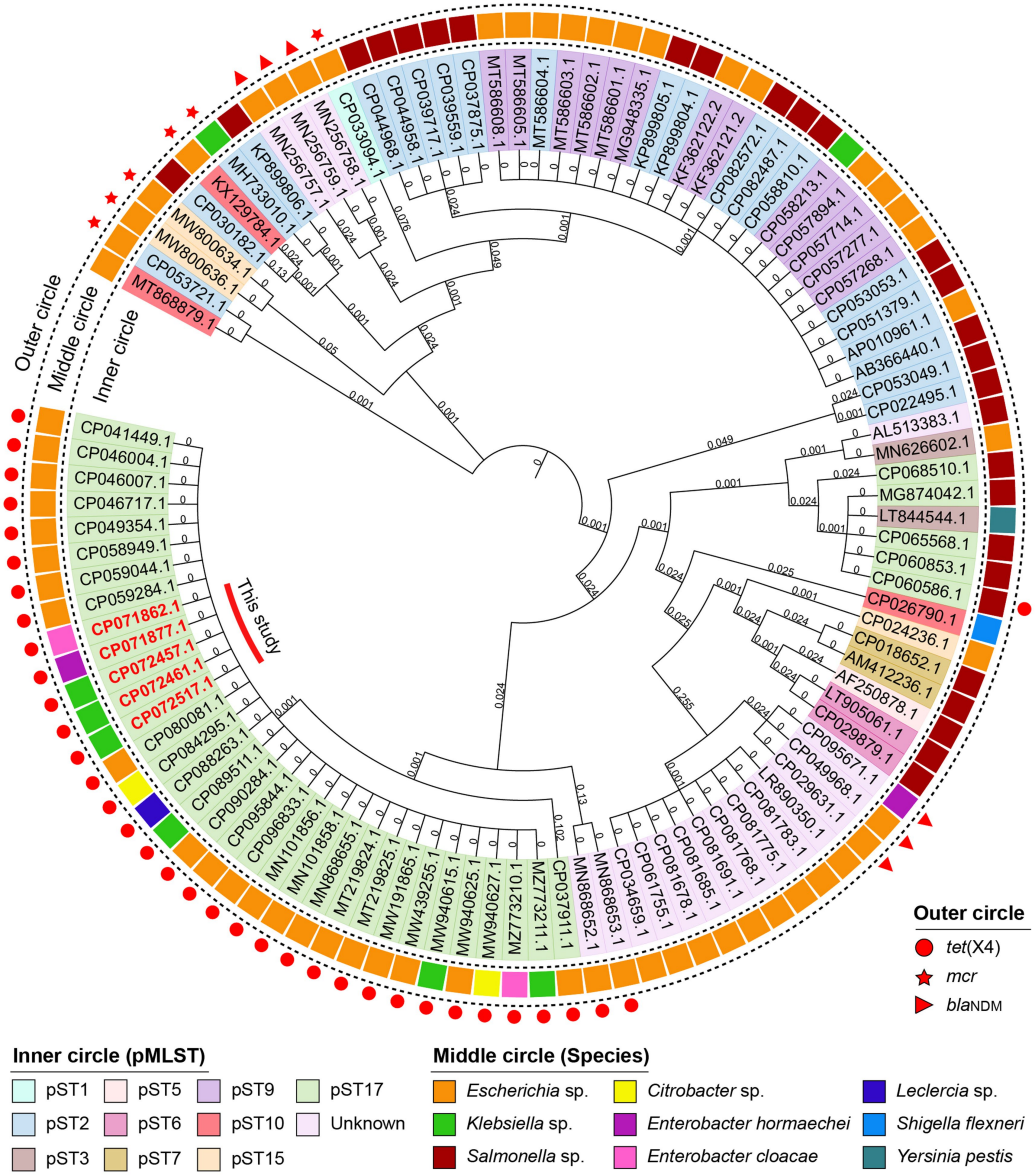
Phylogenetic tree of 100 IncHI1 plasmids with complete sequence from GenBank database. The accession numbers in red are discovered in this study. The innermost to outermost circles indicated the pMLST of plasmids, the species of isolates and the important ARGs on the plasmids.

All strains carried multiple replicons (IncHI1A, IncHI1B and IncFIA), and most *K. pneumoniae* strains carried IncN replicon (Table 1). The complete sequence of strains K1L, 3Z5L, B12L, GX1Z-1 l and GX4-8 l showed that the tet(X4) gene are located on the IncHI1 plasmid and belongs to a novel pMLST. The IncHI1 plasmid was assigned to pST17, which contained two novel alleles, HCM1_043 (4) and HCM1_116 (5). Moreover, we analyzed 100 IncHI1 plasmid sequences from GenBank database that showed all *tet*(X4)-positive plasmids belonged to pST17. Although these plasmids were discovered in different spaces, more commonly *E. coli*, a small amount of *K. pneumoniae* (MW940615), *Salmonella enterica* (CP060586), and *Citrobacter* sp. (MW940627) that have relatively close consanguinity based on the core genes analysis. Similarly, the *mcr* and *bla*_NDM_ positive plasmids have a closer relationship (Figure 2). This suggests that the *tet*(X4), *mcr*, and *bla*_NDM_-positive IncHI1 plasmid in several species were mainly transmitted by cloning.

**Table 1:** The genome characteristics of the *K. pneumoniae*, *E. cloacae* and *Enterobacter hormaechei* strains.

Strains	Species	MLST	Plasmids	Inc type	Size(−kb)	GC content (%)	*tet*(X4) position	Accession no.
3Z5L	*K. pneumoniae*	ST3830	-	-	5,215,453	58	No	CP072515
			p3Z5L-1	IncHI1B	259,774	47.2	No	CP072516
			p3Z5L-2	IncHI1	196,655	46.2	Positive	CP072517
			p3Z5L-3	IncFIB(K)	110,830	52.1	No	CP072518
			p3Z5L-4	IncM2	59,397	51.9	No	CP072519
B12L	*K. pneumoniae*	ST727	-	-	5,419,314	57.3	No	CP072456
			pB12L-1	IncHI1	185,386	46.5	Positive	CP072457
			pB12L-2	IncN	111,105	51.7	No	CP072458
K1L	*K. pneumoniae*	ST727	-	-	5,420,142	57.3	No	CP072460
			pK1L-1	IncHI1-N	301,103	48.3	Positive	CP072461
			pK1L-2	-	9,585	42.0	No	CP072462
GX1Z-1 l	*E. cloacae*	ST524	-	-	4,798,859	54.9	No	CP071861
			pGX1Z-1 l	IncHI1	296,468	48.7	Positive	CP071862
GX4-8 l	*E. cloacae*	ST1862	-	-	4,754,790	55.2	No	CP071876
			pGX4-8 l	IncHI1	195,884	46.2	Positive	CP071877

**Figure 3:**
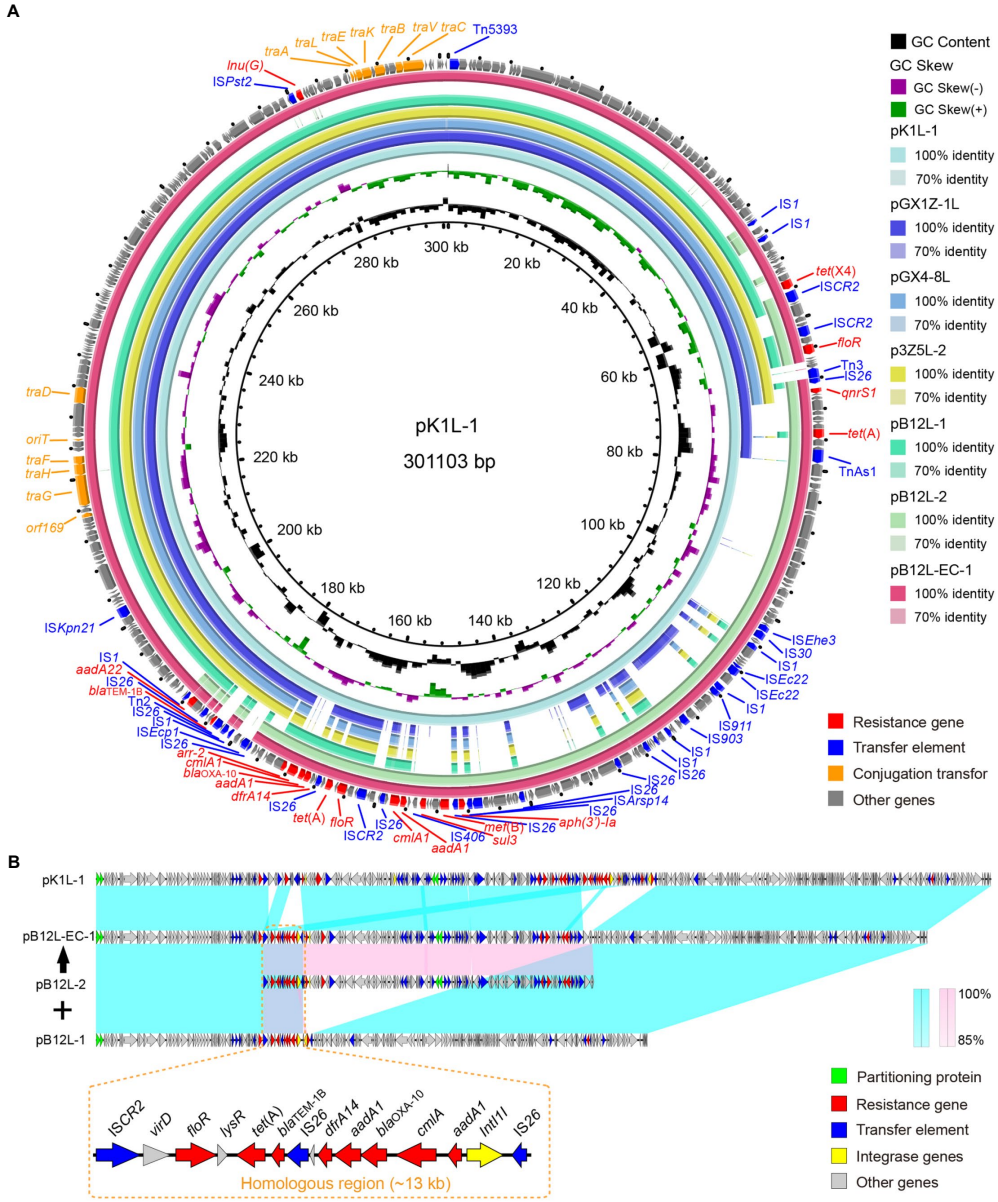
Sequence alignment of *tet*(X) harboring plasmids in this study. **(A)** The circular genetic map of plasmids pK1L, pGX1Z-1 l, pGX4-8 l, p3Z5L-2, pB12L-1, pB12L-2 and pB12L-EC-1. The pK1L is used as the reference. **(B)** Linear comparison of plasmids pK1L, pB12L-1 and pB12L-2 with the transconjugant plasmid pB12L-EC-1. The corresponding alignments are represented in wathet blue and pink. The orange dashed boxes indicated the genes in the homologous region. The arrows and triangles represent genes of different function categories (blue: transfer element; red: AMR gene; green: partitioning protein; golden: integrase and resolvase; gray: other functions).

*Klebsiella pneumoniae* and *E. cloacae tet*(X4) positive plasmids have similar backbone structures (Figure 3A). All the IncHI1 plasmids contained the completed conjugation transfer elements including *oriT*, T4SS and T4CP, but without a conjugation transfer element was identified in IncN plasmid. IncHI1 plasmid-mediated *tet*(X4) transfer risk in different species is underestimated. The core genetic structures of *tet*(X4) remained in the conserved sequence as *abh*-*tet*(X4)-IS*CR2* (Li et al., 2020b). Interestingly, the pK1L (301 kb) is a complex plasmid (IncHI1-N) that is derived by homologous recombination of the IncHI1 (~190 kb) and IncN (~110 kb) from other *K. pneumoniae* strains. The fusion plasmid IncHI1-N was also detected in the transconjugant (pB12L-EC-1) harboring *tet*(X4) from the *K. pneumoniae* B12L (pB12L-1 and pB12L-2) to *E. coli* C600 mediated by IS*CR2* with a ~ 13 kb homologous sequence (IS*CR2*-*virD*-*floR*-*lysR*-*tet*(A)-*bla*_TEM-1B_-IS*26*-*dfrA14*-*aadA1*-*bla*_OXA-10_-*cmlA*-*aadA1*-*IntI1*-IS*26*) (Figure 3B). This is similar to the previously reported that the *mcr-1*-bearing plasmids pD72-mcr1 (IncF33: A-: B-) recombined with pD72-F33 (IncN) that was mediated by IS*26* (He D. et al., 2019). The fusion plasmid pB12L-EC-1 was highly similar to pK1L.

### Conjugation and S1-PFGE analysis of *tet*(X4)-positive strains

**Figure 4:**
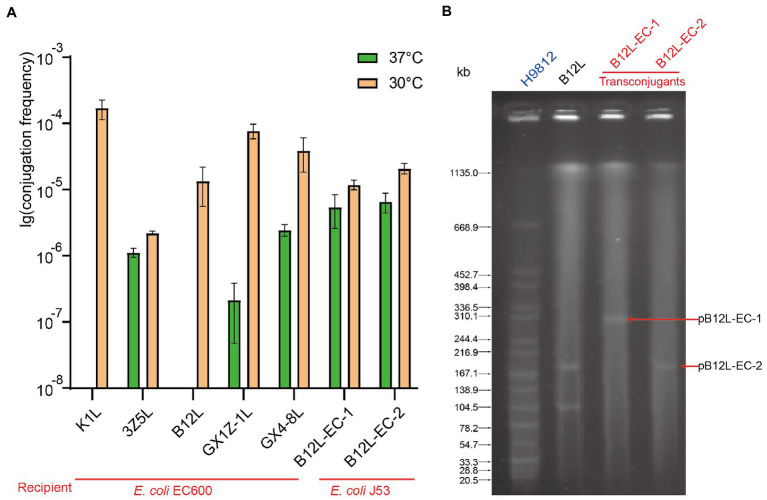
The conjugation transfer frequency of different bacteria and the plasmid profiles of fusion plasmid. **(A)** The conjugation transfer frequency of five *tet*(X4) positive strains and the transconjugants of B12L. **(B)** The S1-PFGE of the donor strain B12L and two types transconjugants (B12L-EC-1 and B12L-EC-2).

The conjugation assay was performed to investigate the transferability of the *tet*(X4) gene in *Enterobacteriaceae*. As with the characteristics of IncHI1 plasmids, conjugation experiments showed that all strains successfully transfer the *tet*(X4) gene to recipient strain *E. coli* C600 with a higher conjugation frequency at 30°C, and a significantly reduced conjugation frequency observed at 37°C (Figure 4A). This is consistent with the characteristics of the thermosensitive plasmids. As mentioned above, the fusion plasmid pB12L-C600-1 containing two replicons, IncHI1 and IncN generated by the conjugation transfer of the *K. pneumoniae* B12L to *E. coli* C600. The plasmid profiles of the donor and transconjugants detected by S1-PFGE showed the two sizes plasmids (about 200 kb and 300 kb) in the transconjugants (Figure 4B).

In order to analyze the ability of conjugation transfer of the IncHI1 and IncHI1-N plasmids. The transconjugants containing the pB12L-EC-1(IncHI1-N) and pB12L-EC-2(IncHI1) were further conjugated transfer to recipient *E. coli* J53 at 30°C or 37°C. The result showed that the pB12L-EC-1 and pB12L-EC-2 had no significant difference in conjugation frequency from EC600 to J53, both at 30°C and 37°C (Figure 4A).

### Antimicrobial susceptibility testing of donors and transconjugants

The *K. pneumoniae*, *E. cloacae* and *E. hormaechei*, as well as their transconjugants were detected the antimicrobial susceptibility. All of the wild-type strains exhibited resistance to AMP, A/C, FFC, TET, TIG, ENR and SUL, and sensitive to CAZ, IMP, MEM and COL. Only one strain GX4-8 l was resistant to GEN. The transconjugants acquired the most of ARGs that have similar resistant profiles (Supplementary Table S1).

## Discussion

The *tet*(X4) is the critical resistant gene for the tetracycline family, especially tigecycline. An increasing number of *tet*(X4)-positive bacteria species have been discovered since it was first discovered in *E. coli* (He T. et al., 2019; Sun et al., 2019). The *E. coli* remains the most common carrier for the *tet*(X4) gene, and the *Citrobacter* sp.*, Acinetobacter sp., K. pneumoniae*, *E. cloacae* and *E. hormaechei* carrying *tet*(X4) gene were reported occasionally. The different bacteria spp. carrying *tet*(X4) gene were always isolated from different farms or regions. Here, we identified the *tet*(X4)-positive *K*. *pneumoniae*, *E. cloacae* and *E. hormaechei* in a pig farm at the same time. These strains carried the same plasmid that belonged to pST17 IncHI1 plasmid. The *tet*(X4)-positive IncHI1 plasmid was also identified in *E. coli* from this farm in our previous study (Feng et al., 2022). It is strong evidence that the IncHI1plasmid mediated the *tet*(X4) gene transfer among different *Enterobacteriaceae*.

The *tet*(X4) gene was always located on the plasmid in *Enterobacteriaceae* and on chromosomes in other bacteria. The IncX1 type plasmid was considered the most common vector for the *tet*(X4) gene, followed closely by the IncHI1 plasmid (Cui et al., 2022). The IncHI1 harbored *tet*(X4) gene is becoming increasingly common in *Enterobacteriaceae* (Fang et al., 2020; Gao et al., 2022). Notice that it has become the most important type for the spread of the *tet*(X4) gene among *Enterobacteriaceae*, except *E. coli*, which has been discovered in *K. pneumoniae* and *E. cloacae*.

pMLST is an important method for tracing the spread of plasmids based on molecular typing. Phan et al. established the typing method for IncHI1 plasmids using variation in six conserved loci (Phan et al., 2009). Seventeen types that have been identified in IncHI1 plasmids and recorded in PubMLST.^3^ The *tet*(X4) gene is only located in the pST17 IncHI1 plasmid, and similar results were observed in *mcr*, *bla*_NDM_ and *bla*_CTX-M_ located in a specific plasmid (Valcek et al., 2021). This suggests that there is some association between resistance genes and plasmid typing. The probability of insertion of resistance genes into plasmids is much lower than plasmid transfer.

The plasmid is the most important vector for transferring the ARGs. The ARGs and the virulence genes are always located on the specific Inc-type plasmids, such as the *mcr-1* mainly in IncX4, IncI2 and IncHI2 plasmid (Rodríguez-Santiago et al., 2021). Hybrid plasmids are becoming increasingly common, which could contribute to the ARGs and virulence genes co-translocation and assist the non-conjugative plasmids transferred (Li et al., 2020a; Tang, M. et al., 2020). The most of hybrid plasmids were derived during the conjugation transfer, and the insertion sequence IS*26* was the most common element for guided the plasmids recombination by the homologous sequence (Liu et al., 2020; Peng et al., 2022). Conversely, the doner plasmids containing the highly similar homologous sequences suggested that the small plasmids may be produced by decomposition of fusion plasmid from other hosts. The mechanism of initial fusion remains to be investigated. Note that the IncHI1 plasmid could remove the temperature restrictions for conjugative transfer by fusion with the IncN plasmid and obtain the resistance and virulence genes at the same time. This study is the first report of the IncHI1 fusion with IncN plasmid that may increase the ability to spread *tet*(X4).

## Conclusion

In summary, we reported the IncHI1 plasmid harboring the *tet*(X4) gene discovered in *K. pneumoniae*, *E. cloacae*, and *E. hormaechei* from the same farm. It provided further evidence that the *tet*(X4) gene transfer among bacteria by IncHI1 plasmid in livestock farm. The *tet*(X4)-positive IncHI1 plasmids belonged to pST17, a novel subtype. Furthermore, the IncHI1 and IncN plasmid tended to fuse by the IS*CR2*, which could increase the risk of co-transfer the ARGs among bacteria. The study highlights that the IncHI1 plasmid is a risk factor for transfer *tet*(X4) among *Enterobacteriaceae*.

## Data availability statement

The datasets presented in this study can be found in online repositories. The names of the repository/repositories and accession number(s) can be found in the article/Supplementary material.

## Author contributions

JM and JW designed the study. BT, HY, MS, and JF collected the samples and conducted the experiments. RL, LB, and YH analyzed and interpreted the data. JM, BT, and ZY drafted the manuscript. All authors contributed to the article and approved the submitted version.

## Funding

This work was supported by the China Postdoctoral Science Foundation [grant number 2021 M702904], China Wool Sheep Industry and Technology System project [grant number CARS-39], the Natural Science Basic Research Plan in Shaanxi Province of China [grant number 2021KW-41], State Key Laboratory for Managing Biotic and Chemical Threats to the Quality and Safety of Agro-products [grant number 2010DS700124-ZZ2008 and 2021DG700024-KF202213], “Leading Goose” R&D Program of Zhejiang Province [2023C03045] and Key Research and Development Program of Zhejiang Province [LY23C180001].

## Conflict of interest

The authors declare that the research was conducted in the absence of any commercial or financial relationships that could be construed as a potential conflict of interest.

## Publisher’s note

All claims expressed in this article are solely those of the authors and do not necessarily represent those of their affiliated organizations, or those of the publisher, the editors and the reviewers. Any product that may be evaluated in this article, or claim that may be made by its manufacturer, is not guaranteed or endorsed by the publisher.

## Supplementary material

The Supplementary material for this article can be found online at: https://www.frontiersin.org/articles/10.3389/fmicb.2023.1128905/full#supplementary-material

## Footnotes

^1^ http://www.genomicepidemiology.org/

^2^ https://rast.nmpdr.org/rast.cgi

^3^ https://pubmlst.org/bigsdb?db=pubmlst_plasmidseqdef
