# Supplementary material for: IncHI1 plasmids mediated the tet(X4) gene spread in Enterobacteriaceae in porcine
Source: Front Microbiol. 2023 Mar 30;14:1128905. doi: 10.3389/fmicb.2023.1128905 (PMC10098456; doi:10.3389/fmicb.2023.1128905)
Supplement: Supplementary file 1 [file Table_1.DOCX]

**Supplementary** **data**

Table S1. Antimicrobial susceptibility testing of *K. pneumoniae*, *E. cloacae* and *E. hormaechei* strains and the transconjugants in this study.

| Strain | Antimicrobial Susceptibility Testing (S, Susceptible; I, intermediate; R, resistant) | | | | | | | | | | | | |
| --- | --- | --- | --- | --- | --- | --- | --- | --- | --- | --- | --- | --- | --- |
|  | AMP | A/C | GEN | FFC | TET | TIG | CEF | CAZ | ENR | SUL | IMP | MER | COL |
| K1L | R | R | S | R | R | R | S | S | R | R | S | S | S |
| K1L-EC | R | R | S | R | R | R | S | S | R | R | S | S | S |
| 3Z5L | R | R | S | R | R | R | S | S | I | R | S | S | S |
| 3Z5L-EC | R | R | S | R | R | R | S | S | S | R | S | S | S |
| B12L | R | R | S | R | R | R | I | S | R | R | S | S | S |
| B12L-EC-1 | R | R | S | R | R | R | S | S | R | R | S | S | S |
| B12L-EC-2 | R | R | S | R | R | R | S | S | S | R | S | S | S |
| GX1Z-1L | R | R | S | R | R | R | S | S | I | R | S | S | S |
| GX1Z-1L-EC | R | R | S | R | R | R | S | S | R | R | S | S | S |
| GX4-8L | R | R | R | R | R | R | I | S | R | R | S | S | S |
| GX4-8L-EC | R | R | S | R | R | R | S | S | R | R | S | S | S |
| 16L | R | R | S | R | R | R | S | S | R | R | S | S | S |
| 29L | R | R | S | R | R | R | S | S | R | R | S | S | S |
| 313L | R | R | S | R | R | R | I | S | R | R | S | S | S |
| 38L | R | R | S | R | R | R | S | S | R | R | S | S | S |
| 39L | R | R | S | R | R | R | I | S | R | R | S | S | S |
| 312L | R | R | S | R | R | R | S | S | R | R | S | S | S |
| 421L | R | R | S | R | R | R | S | S | R | R | S | S | S |
| 423L | R | R | S | R | R | R | S | S | R | R | S | S | S |
| 3Z1L | R | R | S | R | R | R | S | S | R | R | S | S | S |

AMP: ampicillin; A/C, amoxicillin-clavulanate; GEN, gentamicin; FFC, florfenicol; TET, tetracycline; TIG, tigecycline; CEF, ceftiofur; CAZ, ceftazidime; ENR, enrofloxacin; SUL, sulfisoxazole; IMP, imipenem; MER, meropenem; COL, colistin.
